# Baseline depressive symptoms as predictors of efficacy and tolerability of the treatment with duloxetine: a network analysis approach

**DOI:** 10.3389/fpsyt.2023.1210289

**Published:** 2023-06-16

**Authors:** Julian Maciaszek, Tomasz Pawłowski, Tomasz Hadryś, Błażej Misiak

**Affiliations:** Department of Psychiatry, Wrocław Medical University, Wrocław, Poland

**Keywords:** depression, duloxetine, network analysis, depressive symptoms, treatment prediction, adverse drug reaction

## Abstract

**Introduction:**

Depression is considered one of the most prevalent and burdensome mental disorders. Only 50–60% of patients respond to first-line treatment. Individuals with depression might benefit from personalized treatment, tailored to the individual needs of the patient. In this study, we aimed to explore the baseline characteristics of depressive symptoms associated with a good response to duloxetine treatment using a network analysis. Additionally, the relationship between baseline psychopathological symptoms and treatment tolerability was assessed.

**Methods:**

The sample of 88 drug–free patients with active depressive episode, who started monotherapy with increasing doses of duloxetine were evaluated. The Hamilton Depression Rating Scale (HAM-D) was used to assess depression severity and the UKU side effect rating scale to monitor adverse drug reactions (ADRs). A network analysis that explored interactions of specific baseline depression symptoms, treatment efficacy and tolerability was performed.

**Results:**

The node representing duloxetine treatment efficacy was directly connected to the nodes representing the first HAM-D item (“depressed mood”) (edge weight = 0.191) and duloxetine dose (edge weight = 0.144). The node representing ADRs was directly connected to only one node representing the baseline score of the HAM-D anxiety (psychic) item (edge weight = 0.263).

**Discussion:**

Our findings indicate that individuals with depression presenting greater levels of depressed mood and lower levels of anxiety symptoms might better respond to the treatment with duloxetine in terms of efficacy and tolerability.

## 1. Introduction

It has been estimated that about 280 million people suffer from depression worldwide, and thus it is considered one of the most prevalent and burdensome mental disorders (1). Indeed, depression is also the second leading cause of disability, which appears to exert a major economic burden, since it largely affects people in working age (2). Severe depression has direct consequences as about 60% of people who commit suicide suffer from depression (3). Despite a great progress in the availability of evidence-based therapies of depression, a large proportion of patients do not respond to treatment or suffer from side effects (4). Only 50–60% of patients with major depression disorder (MDD) respond to the first-line treatment, and only 35–40% of patients achieve remission of symptoms during the first 8 weeks of treatment (5). There is also a high interindividual variability in responses to antidepressant treatment in terms of specific pharmacotherapies and effective dosage of specific antidepressants. Accumulating evidence indicates that individuals with MDD might benefit from personalized treatment, tailored to the individual needs of the patient. However, translation of personalized treatments to clinical practice still requires better understanding of the underlying mechanisms as well as the development of reliable predictors of clinical and functional outcomes.

Notably, the pooled analysis of four clinical trials conducted in China revealed that the initial clinical symptom presentation may have a crucial impact on the prognosis of treatment with escitalopram (6). In the sample of 649 MDD outpatients, it was observed that individuals without accompanying anxiety symptoms show the greatest improvements on the 17-item Hamilton Depression Rating Scale (HAM-D) during the treatment with escitalopram (6). Another meta-analysis showed that baseline depressive symptoms clusters related to somatic symptoms, such as the “sleep/sexual/somatic” cluster and the “gastrointestinal/weight loss” cluster, are associated with better response to duloxetine treatment in comparison with placebo (7). Other studies revealed that baseline symptoms represented by psychomotor retardation, executive dysfunction, and hopelessness might be associated with negative outcomes of treatment with antidepressants (8), whereas higher baseline suicidality was associated with a better treatment response (9). However, other reports suggest that this association is not significant or that higher baseline suicidality may be associated with a worse treatment response (10, 11). Following these considerations, it should be noted that the symptom profile of depressed patients show significant heterogeneity that is reflected in a wide range of MDD subtypes (e.g., melancholic, atypical, and psychotic depression) (12). It has been shown that several neural pathways may correlate with certain psychological and physical symptoms of MDD. This heterogeneity of clinical manifestation is most likely the result of alterations in various aspects of normal neural functions that can range from the molecular level to the level of neural circuits (13). There may exist several subtypes of MDD with different underlying mechanisms (14). Consequently, various approaches that address clinical heterogeneity, e.g., those related to the use of clustering methods have been proposed. It seems that the utility and practicability of findings from these studies might be much more easily used in everyday clinical practice in comparison with those based on various biomarkers due to their high cost, and limited availability. These observations point to the rationale of investigating clinical predictors of treatment response in order to facilitate the development of personalized approaches.

Duloxetine is an antidepressant representing serotonin and noradrenalin reuptake inhibitors (15) that exerts some affinity to other receptors, including muscarine, noradrenaline, dopamine and histamine receptors (16). Although the previous meta-analysis (7) showed that somatic symptoms at baseline are associated with better response to duloxetine treatment in comparison with placebo, another pooled analysis of data from 10 randomized control trials comparing the efficacy of duloxetine (40–60 mg/day) over placebo data from showed that early improvement of retardation symptoms may serve as a modest predictor of remission at the follow-up (17). Also, Tokuoka et al. (18) compared duloxetine remitters and non-remitters showing a tendency for the greatest improvement by duloxetine in those showing the highest depression scores at baseline.

Studies investigating clinical predictors of response to treatment in MDD are often based on analytical approaches that use composite scores of various symptom clusters. Consequently, insights into the relevance of single symptoms might be limited. However, specific methods associated with the network theory enable the exploration of dynamic interactions among individual indicators or their dimensions with other factors, such as treatment outcomes. A network analysis presents a dynamic implementation of depressive symptoms that interact with each other to produce a specific phenotype. In this context, connections between symptoms (i.e., nodes) are referred to as edges, and disorders are conceptualized as patterns of dynamic relationships between symptoms, whereby greater connectivity corresponds to an increased vulnerability to psychopathology (19). Network models concentrate on evaluating the distinct variance between various indicators. This idea makes it simpler to comprehend the diverse nature of MDD, which is composed of a variety of symptoms.

Although convincing evidence suggests the association of baseline symptoms profile with antidepressant treatment efficacy, certain research gaps regarding specific antidepressants still exist. It remains unknown what characteristics of symptoms are most closely associated with duloxetine treatment outcomes. On the other side, it remains unknown as to whether any specific symptom profile is most closely associated with adverse drug reactions (ADRs) related to the treatment with duloxetine. In this regard, the present study aimed to explore the baseline characteristics of depressive symptoms associated with a good response to duloxetine treatment using a network analysis. Additionally, we aimed to assess the relationship between the network of baseline psychopathological symptoms and treatment tolerability. To address this aim, we used a network analysis that has explored interactions of specific baseline depression symptoms, treatment efficacy and tolerability. To our knowledge, a network analysis of factors predicting response to treatment with duloxetine has not been performed so far.

## 2. Materials and methods

**FIGURE 1:**
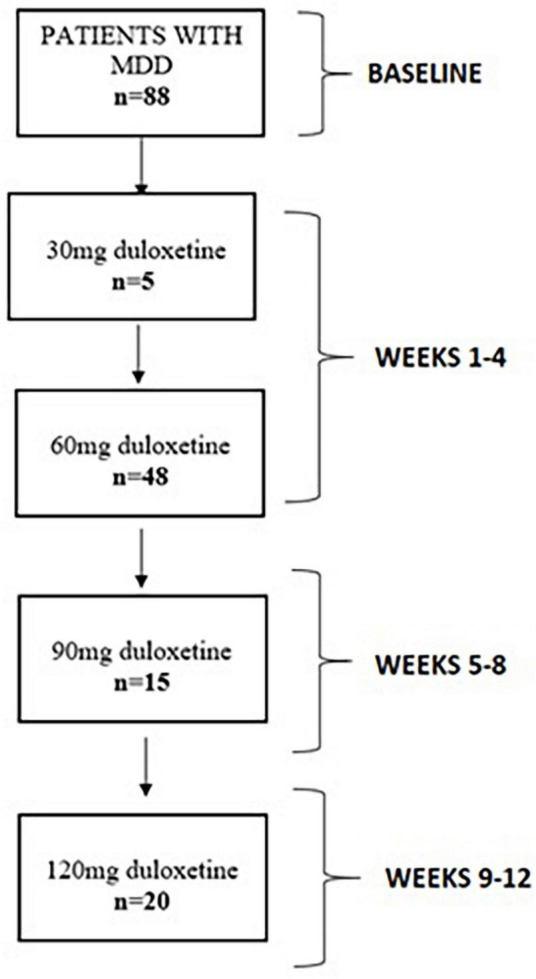
Flow diagram of study participants.

The present study was based on a sample of 88 drug–free patients with active depressive episode, who started monotherapy with increasing doses of duloxetine in accordance to the protocol. This study was performed as a part of the larger project investigating the efficacy and tolerability of duloxetine with respect to gene polymorphisms. The patients did not take additional psychiatric drugs. The study was conducted at the outpatient clinic (Department of Psychiatry, Wrocław Medical University, Wrocław, Poland). Participants were included if they met the following inclusion criteria: (1) age between 18 and 85 years; (2) no current psychiatric treatment and (3) a diagnosis of MDD based on the ICD-10 criteria, which lasted at least 14 days. Patients were excluded from the study group based on the following exclusion criteria: (1) coexisting diagnosis of schizophrenia; (2) bipolar disorder; (3) dementia; (4) substance use disorders (except of nicotine dependence); (5) severe or unstable somatic disease and (6) non-compliance with duloxetine monotherapy. The patients were examined every 4 weeks by the psychiatrist who decided, on the basis of clinical manifestation, to initiate treatment with duloxetine according to the schedule presented in the flow diagram (Figure 1): (1) baseline assessment: the recommendation to take 30 mg over the period of 2 weeks, followed by the increase of the dosage to 60 mg once daily; (2) assessment after 4 weeks from baseline and increase of the dosage to 90 mg if needed; (3) assessment after 8 weeks from baseline and increase of the dosage to 120 mg if needed and (4) follow–up assessment after 12 weeks from baseline. The duloxetine dose was increased according to MDD treatment guidelines (20, 21), by 30 mg at the following time points for patients who failed to achieve remission, i e., they did not achieve the HAM-D total score of ≤ 7 (22). Reassessment visits were terminated if further dosage increase appeared to be not necessary. The study was approved by the Ethics Committee at Wrocław Medical University (Wrocław, Poland, approval number: 606/2017).

The severity of depressive symptoms was assessed using the Hamilton Depression Rating Scale (HAM-D) (23). The HAM-D is a 21-item questionnaire used to measure the severity of depressive symptoms, with a focus on somatic symptoms and anxiety. The UKU Side Effect Rating Scale (UKU SERS) containing 48 items was used for recording ADRs in the preceding 3 days (24). This scale requires to be used for a duration of 3 days. Each item is scored on a 4-point scale (possible responses vary between 0–“no, not at all” and 3–“much more than usual.” The ADRs were documented based on the feedback obtained from the patients on each of the parameters listed in the UKU SERS scale. The items were clustered into four sub-groups: psychic, neurological, autonomic and other side effects. After each clinical assessment, it was indicated if specific ADRs were attributable to the treatment with duloxetine (25). The total UKU-SERS score was the sum of points for the ADRs attributable to the treatment with duloxetine. Additional measures included the information about age, sex, the level of education, marital status, age of MDD onset, chronic somatic conditions, and vocational status.

Data were analyzed by means of the network analysis. A network analysis of the characteristics of depressive symptoms at baseline expressed as: (1) 21 items of the HAM-D; (2) depressive treatment efficacy expressed as the percentage of the HAM-D reduction from baseline to the end point; (3) duloxetine dose in the end point of the treatment, treatment time (in months) and; (4) duloxetine tolerability expressed as the total UKU-SERS score at the end point of the treatment was carried out. Network estimation was performed using the “EBICglasso” model (26). This approach allows to use ordinal and continuous variables. The Extended Bayesian Information Criterion (EBIC) was used to select the penalty parameter, which is a tuning parameter that controls the level of sparsity. This was based on prior research by Foygel and Drton (27). In this study, the parameter was set at 0.5, as recommended by Epskamp and Fried (28). The network that resulted from this analysis included 21 depressive symptoms from the HAM-D, the percentage of HAM-D reduction, treatment time, duloxetine dose, and the sum of ADRs, all of which were connected with edges. The thickness of the edges represented the strength of the association between the nodes, with thicker nodes indicating stronger associations. The central variables (nodes) were identified by analyzing the node strength, which is the sum of all edge weights connected to the node. This is a commonly used indicator of centrality (27, 29, 30). The accuracy and stability of the network were analyzed using the case-drop bootstrap procedure with 1,000 iterations, which was used to assess the stability of the node strength (31). The stability of the node strength was visualized and estimated using the correlation stability coefficient (CS-C), which should be higher than 0.25. Additionally, the non-parametric bootstrap procedure with 1,000 iterations was used to analyze the 95% confidence interval (CI) of edge weights. A greater 95%CI corresponds to lower precision in the estimation of edge weights. Data analysis was performed using the JASP software.

## 3. Results

### 3.1. General characteristics

The general characteristics of the sample are shown in Table 1. The mean age of the study participants was 38.3 ± 16.5 years. Participants were more likely to be females (59.1%), persons with a higher level of education (65.9%), and employed individuals (44.5%). The mean time from the first depressive episode was 3.9 ± 7.1 years, 39 (44.3%) patients suffered from initial and 49 (55.7%) from recurrent depression. The mean HAM-D baseline score was 19.7 ± 7.8 and the mean reduction of the HAM-D depressive symptoms from the baseline to the end point was 59.2 ± 38.9%. The mean of the total UKU SERS score was 6.8 ± 7.6 points, 5 patients discontinued therapy due to a severe adverse drug reaction. Supplementary Table 1 shows the occurrence of adverse drug reactions depending on the dose of duloxetine. A total of 53 patients completed the treatment after 1 month; 5 of them kept 30 mg, 48 completed the treatment protocol with the daily dose of 60 mg; 15 patients completed the treatment after 2 months taking 90 mg of duloxetine daily; 20 patients completed the treatment after 3 months taking 120 mg of duloxetine daily (Figure 1).

**TABLE 1:** General characteristics of the sample.

Variable	Mean ± SD or n (%)
Age	38.3 ± 16.5
Sex (female)	52 (59.1%)
Marital status (married)	26 (29.5%)
Education (higher education)	58 (65.9%)
Work (working)	48 (54.5%)
Dwelling-place (urban)	82 (92.9%)
Education years	15.1 ± 2.8
Somatic conditions*	26 (29.5%)
Illness duration (years)	3.9 ± 7.1
Recurrent depression	49 (55.7%)
UKU SERS	6.8 ± 7.6
HAM-D (baseline score)	19.7 ± 7.8
HAM-D (reduction, %)	51.3 ± 38.9

Data expressed as n (%) or mean (SD).

*Detailed frequency of comorbid somatic diseases in the study group expressed as n (%) is as follows: diabetes mellitus–5 (5.7%); hypertension –12 (13.6%); asthma–6 (6.8%); chronic obstructive pulmonary disease–4 (4.5%), coronary artery disease–3 (3.4%); rheumatoid arthritis–2 (2.3%); chronic kidney disease–2 (2.3%); chronic migraines–8 (9.1%), osteoarthritis–5 (5.7%); chronic back pain–7 (8.0%), and other–5 (5.7%).

### 3.2. Network structure

**FIGURE 2:**
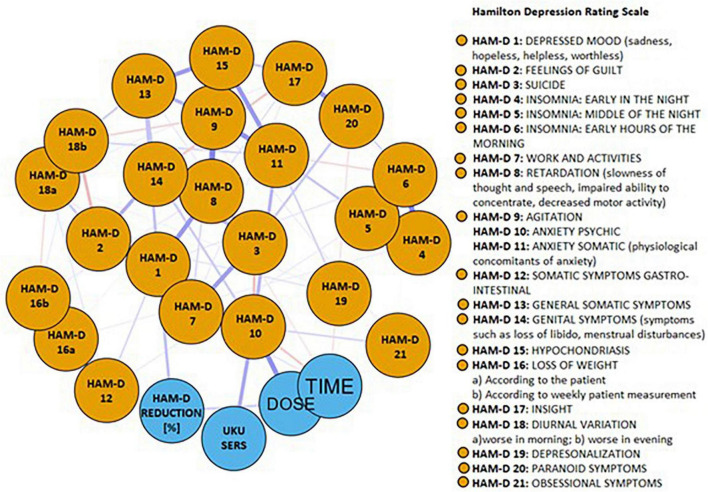
The network of baseline depressive symptoms, duloxetine efficacy, adverse drug reactions, treatment time and duloxetine dose. Blue edges represent positive, while pink edges represent negative correlations between nodes.

The network of baseline depressive symptoms, duloxetine efficacy, ADRs, treatment time and duloxetine dose is shown in Figure 2. Blue edges represent positive, while pink edges represent negative correlations between nodes. Specific groups of nodes appeared to be well-connected, and only a few negative edges were found. Out of 351 edges, weights of 74 edges (21.1%) were higher than zero (Supplementary Table 2). The node representing duloxetine treatment efficacy was directly connected to the nodes representing the first HAM-D item (“*depressed mood*”) (edge weight = 0.191) and duloxetine dose (edge weight = 0.144). The node representing ADRs was directly connected to only one node representing the baseline score of the HAM-D “*anxiety (psychic)*” item (edge weight = 0.263).

### 3.3. Central nodes

**FIGURE 3:**
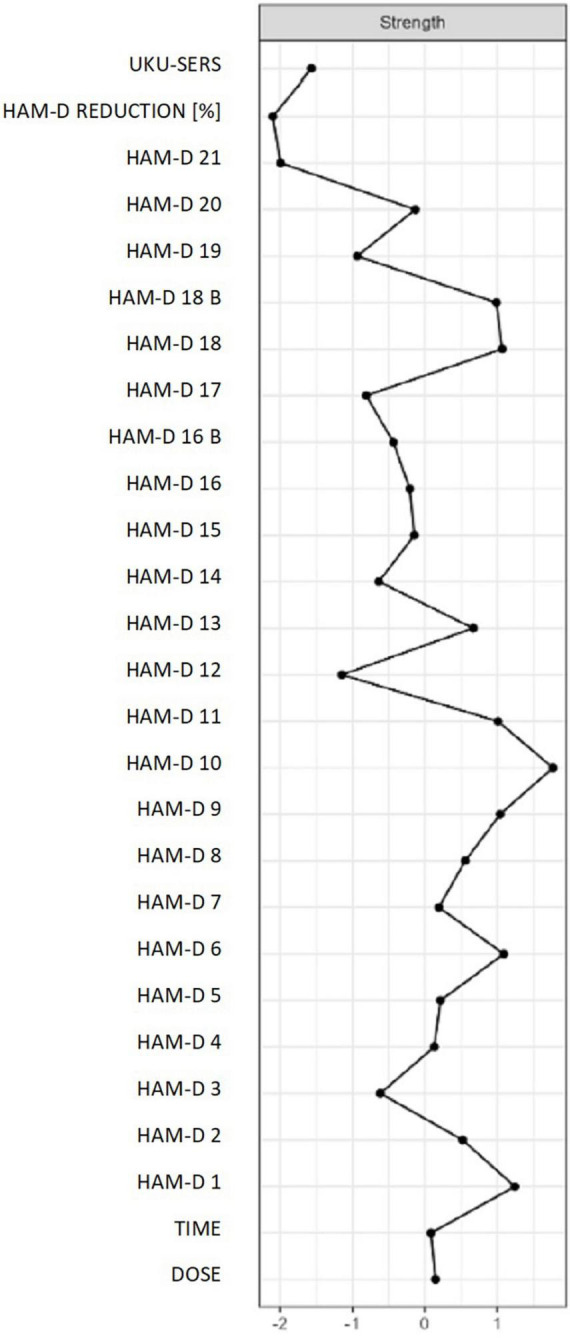
Network centrality values.

The network centrality values are shown in Figure 3. The highest network centrality values were obtained for the following nodes: (1) the HAM-D item 10–“*anxiety (psychic)*” (strength = 1.7); (2) the HAM-D item 9–“*agitation*” (strength = 1.5); (3) The HAM-D item 18b–“*daily swings*” (strength = 1.3); (4) the HAMD-D item 1–“*depressed mood*” (strength = 0.8) and (5) the HAM-D item 11 – “*anxiety somatic*” (strength = 0.7). Results of the analysis testing for between-node differences in the strength, betweenness and closeness centrality index are illustrated in Supplementary Figure 1.

### 3.4. Network stability and accuracy

The node-specific strength appeared to be stable when dropping various proportions of data (Supplementary Figure 2). The bootstrapped 95%CI ranges of edge weights were relatively narrow suggesting sufficient accuracy (Supplementary Figure 3).

## 4. Discussion

To our knowledge, the present study is the first to use a network analysis to investigate the associations of specific depressive symptoms at baseline with the efficacy and tolerability of duloxetine. The network stability and accuracy were satisfactory and all nodes appeared to be well-connected. Findings from our network analysis indicate that a higher severity of depressed mood at baseline might be the only direct symptom predictor of greater duloxetine efficacy whereas the level of anxiety symptoms was the only direct symptom predictor of ADRs during duloxetine treatment. This could indicate that duloxetine might be more effective and better tolerated among patients with higher levels of depressed mood, and lower levels of anxiety.

Our observations are partly in line with those obtained by Tokuoka et al. (18) who found that higher baseline scores of the HAM-D item 1 “*depressed mood*” might be associated with the greatest improvement among duloxetine responders. This observation is in agreement with our results also pointing that the HAM-D item 1 “*depressed mood*” node was associated with a greater HAM-D reduction at the end of the treatment. On the other hand, our results differ from the results obtained in a meta-analysis of duloxetine trials (7) where the largest effect size regarding treatment efficacy was found in the clusters with severe somatic symptoms such as the “sleep/sexual/somatic” cluster, and the “gastrointestinal/weight loss” cluster.

At this point, it is important to note that a network analysis differs methodologically from other approaches to analyze the data as it takes into consideration the effects of several variables that are included in the network. In the pursuit of comprehending the predictive value of the depressive mood item, it is essential to discuss its connections with other HAM-D nodes. These interconnected nodes may indirectly influence the reduction of depressive symptoms. Notably, the nodes representing HAM-D items 2, 7, and 8, which pertain to the axial symptoms of depression encompassing guilt, retardation, as well as work and activity impairment, showed the most substantial indirect influence through their connectivity with the HAM-D item 1 node. These findings align entirely with the previously discussed outcomes obtained by Tokuoka et al. (18). Furthermore, the HAM-D item 1 was also connected with other nodes, i.e., the HAM-D items 12, 13, and 6, which refer to general somatic symptoms, somatic symptoms originating from the gastrointestinal tract, and insomnia, respectively. This interconnection might also be in line with the results obtained by Schacht et al. (7), who demonstrated the primary predictive role of *sleep/sexual/somatic* clusters and the *gastrointestinal/weight loss* clusters with respect to antidepressant treatment efficacy of duloxetine. By considering these interconnected nodes, a more comprehensive understanding of the predictive value of the HAM-D depressive mood 1 item, as well as its broader implications for the reduction of depressive symptoms including insomnia, somatic symptoms and axial symptoms of depression, can be attained.

In our study, the node representing duloxetine treatment efficacy was also directly connected to the node representing duloxetine dose what means that a higher dose was related to better treatment efficacy. These results correspond to those obtained by the pharmacokinetic study (32), where a significant curvilinear quadratic relationship between the improvement of depression scores and plasma duloxetine levels was found. In this study, the incidence of anxiety or irritability was associated with the highest plasma level of duloxetine which could be perceived as ADRs, whereas in our study there were no connections between the dose of duloxetine and the reduction of the HAM-D score. Authors of this study suggested an optimal anxiolytic efficacy of duloxetine at intermediate plasma levels. In another study performed by Polychroniou et al. (33), patients treated with either duloxetine or escitalopram experienced a similar mean number of overall side effects and did not differ in terms of the profile of the specific side effects. Interestingly, in this study duloxetine-treated patients who experienced dry mouth were significantly more likely to achieve remission than those who did not.

## 5. Limitations

There are certain limitations of the present study. Our sample was relatively small in comparison with other network analysis studies; however, recent studies show a tendency to accept smaller samples in studies approaching this methodology (34–37). The lack of a double-blind randomized clinical trial design can be perceived as another limitation. Also, we did not include any measures of treatment adherence. Finally, it is important to note that the network structure might depend on the inclusion of specific variables. For instance, we did not record certain characteristics that might be associated with treatment efficacy (e.g., personality traits, treatment adherence, the level of illness insight) that might impact observed connections. It is also important to note that individuals with first-episode depression and those with recurrent depression might differ in terms of responses to antidepressants. This point was not addressed in the present study due to low sample size. Finally, limited duration of the study does not provide insights into long-term predictors of response to treatment with duloxetine.

## 6. Conclusion

In sum, our findings indicate the associations of the HAM-D item 1–“*depressed mood*”) with greater duloxetine treatment efficacy, and the association of the HAM-D item 10–*anxiety (psychic)* with worse duloxetine tolerability. In other words, our study can be concluded that duloxetine is more effective and better tolerated among patients with dominant depressive mood symptoms and low levels of coexisting anxiety symptoms. These observations might hold some promise for personalizing treatment approaches in people with MDD. However, certain directions for future studies need to be indicated before translation of findings into clinical practice. Importantly, future studies need to replicate our findings in larger samples with longer observation periods using the double-blind RCT designs. Also, it is needed to test baseline predictors of response to duloxetine in people with anxiety disorders in order to better understand the impact of depressive and anxiety on treatment outcomes.

## Data availability statement

The raw data supporting the conclusions of this article will be made available by the authors, without undue reservation.

## Ethics statement

The studies involving human participants were reviewed and approved by the Ethics Committee at Wrocław Medical University (Wrocław, Poland) 50-367 Wrocław, ul. J. Mikulicza-Radeckiego 4a (approval number: 606/2017). The patients/participants provided their written informed consent to participate in this study.

## Author contributions

JM and BM: conceptualization, methodology, software, and writing–original draft. TH: validation, investigation, and visualization. TP: formal analysis, resources, data curation, and supervision. BM: writing–review and editing. JM: project administration. JM and TP: funding acquisition. All authors have read and agreed to the published version of the manuscript.
