# Supplementary material for: Baseline depressive symptoms as predictors of efficacy and tolerability of the treatment with duloxetine: a network analysis approach
Source: Front Psychiatry. 2023 Jun 16;14:1210289. doi: 10.3389/fpsyt.2023.1210289 (PMC10312095; doi:10.3389/fpsyt.2023.1210289)
Supplement: Supplementary file 1 [file Table_1.DOCX]

| **­**Dose [mg] | Total n (%) | | | Psychic | | Neurological | | Autonomic | | Other | |  |  |  |  |  |
| --- | --- | --- | --- | --- | --- | --- | --- | --- | --- | --- | --- | --- | --- | --- | --- | --- |
| **30** |  | 5 (5.7%) | 5 (5.7%) | | 1 (1.1%) | | 3 (3.4%) | | 2 (2.3%) | |  | |  | |  | |
| **60** |  | 29 (33.0%) | 17 (19.3%) | | 14 (15.9%) | | 21 (23.9%) | | 14 (15.9%) | |  | |  | |  | |
| **90** |  | 10 (11.4%) | 7 (8.0%) | | 5 (5.7%) | | 7 (8.0%) | | 6 (6.8%) | |  | |  | |  | |
| **120** |  | 16 (18.2%) | 11 (12.5%) | | 11 (12.5%) | | 13 (14.8%) | | 11 (12.5%) | |  | |  |  | |  |

**Supplementary Table S1.** The occurrence of adverse drug reactions depending on the dose of duloxetine

*Data expressed as n (%)*
