# Supplementary figures and images for: Baseline depressive symptoms as predictors of efficacy and tolerability of the treatment with duloxetine: a network analysis approach
Source: Front Psychiatry. 2023 Jun 16;14:1210289. doi: 10.3389/fpsyt.2023.1210289 (PMC10312095; doi:10.3389/fpsyt.2023.1210289)

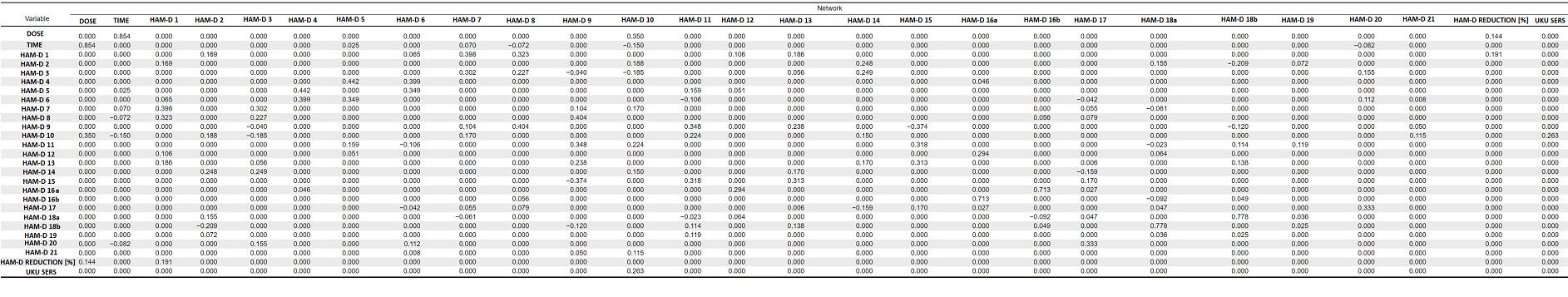

Supplement: Supplementary file 2 [file Image_1.JPEG]

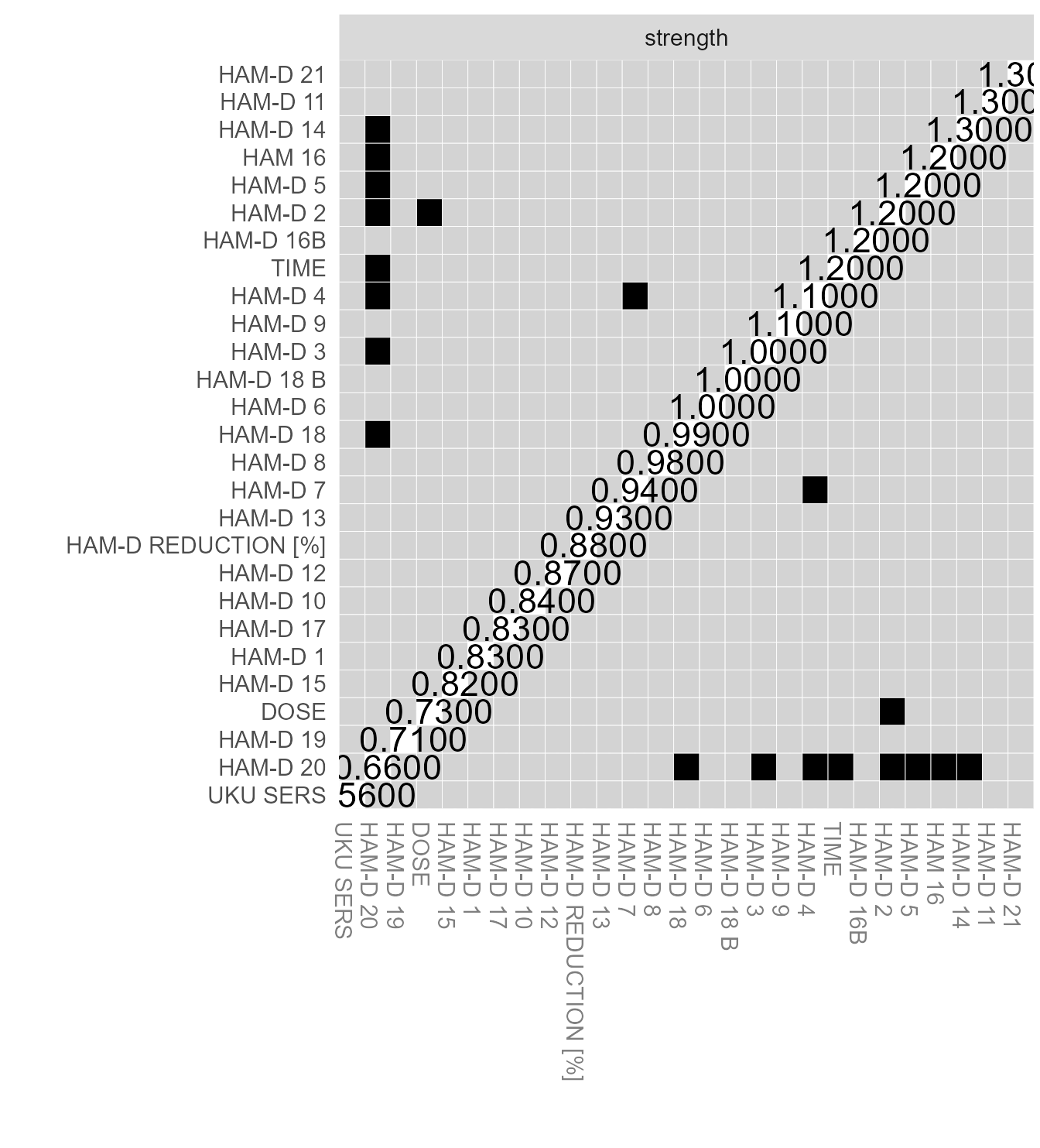

Supplement: Supplementary file 3 [file Image_2.TIFF]

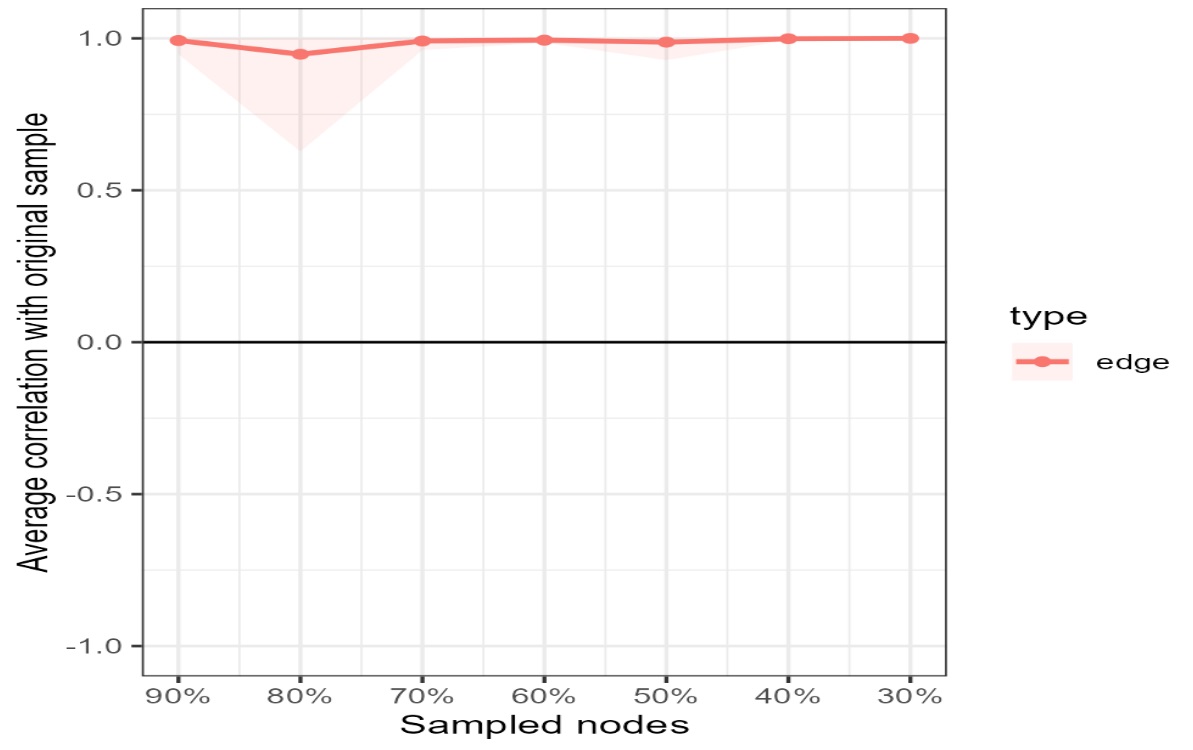

Supplement: Supplementary file 4 [file Image_3.JPEG]

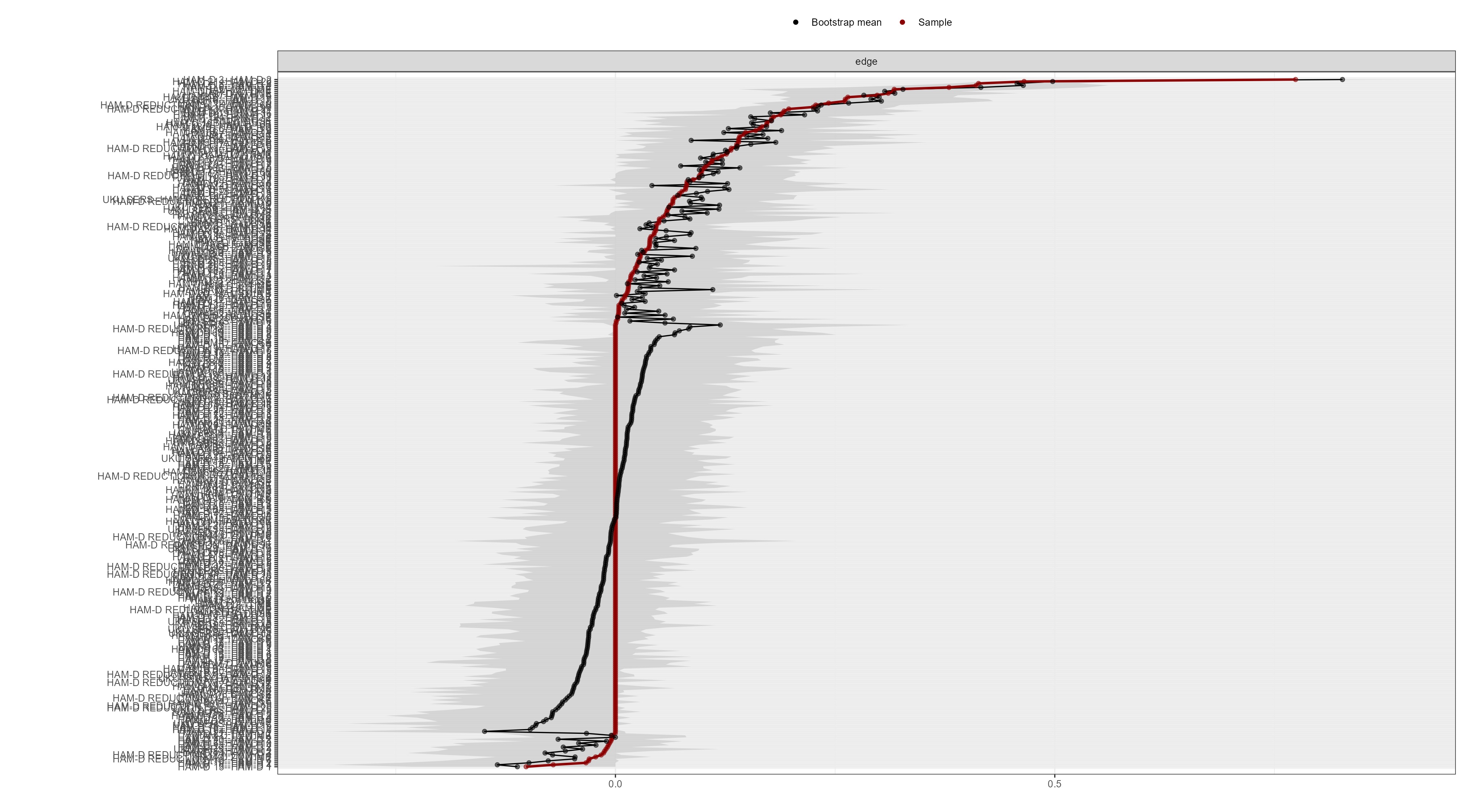

Supplement: Supplementary file 5 [file Image_4.JPEG]
